# Vertical Drainage Performance of a Novel Anti-Clogging Plastic Vertical Drainage Board for Soda-Residue-Stabilized Soil

**DOI:** 10.3390/ma18245661

**Published:** 2025-12-17

**Authors:** Aiwu Yang, Tianli Liu, Ridong Fan, Hao Zhang, Fayun Liang, Xuelun Liu, Guowei Song

**Affiliations:** 1 Department of Civil Engineering, College of Environmental Science and Engineering, Donghua University, Shanghai 201620, China; 2 Department of Geotechnical Engineering, College of Civil Engineering, Tongji University, Shanghai 200092, China; 3 Tianjin Haibin Geotechnical Engineering Co., Ltd., Tianjin 300384, China

**Keywords:** soda-residue-stabilized soil, prefabricated vertical drain (PVD), hydraulic conductivity, vacuum consolidation, indoor model test

## Abstract

In the treatment of soda-residue-stabilized soil with high water content using drainage boards with vacuum preloading, the boards often prone to clogging and bending under lateral pressure, reducing their hydraulic conductivity and affecting the soil reinforcement. In this study, the structure of the standard plastic drainage board (filter membrane + filter core) was improved, and three types of new anti-clogging plastic drainage boards with different structures were developed (Type X: geotextile + filter core, Type Y: geotextile + wire mesh + filter core, Type Z: geotextile + filter membrane + filter core). Permeability tests were subsequently used to determine the optimal structure. In-lab vertical draining tests with vacuum preloading were carried out on the selected model to study the change in water content, vacuum pressure, surface settlement, vane shear strength, and pore water pressure of soil with drainage board insertion depth, providing a reference for the application of new anti-clogging drainage boards in engineering. The results showed that: (1) the type Y anti-clogging plastic drainage board (geotextile + wire mesh + filter core) exhibits the most balanced performance in terms of permeability, anti-clogging ability, tensile strength and bending strength and is suitable for vacuum preloading of soda residue with high water content; (2) the mechanical properties and anti-clogging performance of drainage boards are highly dependent on their structural configuration. Introducing a wire mesh between the filter core and the geotextile significantly enhances the tensile and bending strength of the drainage board without noticeably compromising its drainage performance; (3) the insertion depth of the drainage board significantly affects drainage efficiency, vacuum transmission rate, and strength development of the soda residue. The effective reinforcement range of the drainage board is not limited to the insertion depth but also extends below the bottom of the drainage board.

## 1. Introduction

Soda residue is a waste material generated during the industrial production of soda ash, characterized by naturally high water content, low permeability, and high compressibility [1,2]. However, its comprehensive utilization rate is relatively low [3]. Notably, soda residue treated with vacuum preloading and draining can be used as artificial soil or building material [2]. The plastic drainage boards used in the vacuum preloading process consist of a filter membrane and filter core; however, these boards are prone to clogging and exhibit unsatisfactory bending resistance. During the treatment of high-water-content soil, clogging can occur, reducing the permeability of the drainage board and affecting the effectiveness of vacuum preloading [4]. Critically, soda-residue-stabilized soil is facing challenges due to the application of standard plastic drainage boards with vacuum preloading in high water content.

Researchers have assessed soda-residue-stabilized soil, including the optimal ratio of soda residue in soil, the physical and mechanical properties of the modified soil, its strength development mechanism, and application in road engineering. In terms of the optimal soda residue ratio and the physical and mechanical properties of soda-residue-stabilized soil, most study results have indicated that higher blending ratios may be inferior, instead promoting a peak value (approximately 30–35%). The addition of appropriate amounts of soda residue may improve the unconfined compressive strength, shear strength, and water stability of soil, with excessive soda residue negatively affecting the physical and mechanical properties of soil [5,6,7,8]. Among different studies, the values of the optimal blending ratio of soda residue were not always consistent and greatly affected by differences in soil conditions. In some studies, the soda residue blending ratio needed to achieve the optimal soil state was approximately 15% [9]. In terms of the physical and mechanical properties of soda-residue-stabilized soil under special loading conditions, some researchers have conducted laboratory tests on the strength characteristics and pore water pressure changes in soil under cyclic loading. The results showed that under these conditions, soda-residue-stabilized soil exhibited a clear thixotropy. After resting for 5 days, the strength of the soil was still reduced by about half after four vibration-rest cycles. As a result, cyclic loading was found to significantly affect the cumulative pore pressure of the soil, where the greater the dynamic stress ratio, the greater the cumulative pore pressure under the same number of cycles [10,11]. Regarding the strength development mechanism of soil, studies found that an increase in soil compaction, compressive strength, and shear strength caused by soda residue was due to physical changes and chemical reactions. The physical changes mainly included pore filling and cementation effects, while the chemical reactions included ion exchange, crystallization, pozzolanic reaction, carbonation, and the formation of crystalline hydrates (i.e., ettringite). The decrease in compressive strength caused by excessive soda residue was mainly due to the high water content and low density of the residue, preventing soil densification, and the excessive expansion of ettringite [8,9,12,13]. In terms of the application of soda-residue-stabilized soil in road engineering, some researchers prepared modified soil by mixing soda residue and fly ash in different proportions and added lime to the soil to improve its bearing capacity when used as a subgrade material. The researchers measured the cohesion, internal friction angle, and unconfined compressive strength of the soil with different fly ash contents. The results showed that adding fly ash to soda-residue-stabilized soil improved the cohesion, internal friction angle, unconfined compressive strength, and water stability of soil, while the addition of lime improved its bearing capacity, compression modulus, and compaction [14,15]. These studies indicated that an appropriate amount of soda residue effectively stabilized the soil and improved its physical and mechanical properties. However, the thixotropy of the modified soil and the adverse effects of cyclic loading on its physical and mechanical properties cannot be ignored. As a result, soda residue must be treated through physical methods, such as using drainage boards combined with vacuum preloading to reduce its water content for reinforcement, or chemical methods (i.e., mixing lime or other curing agents into soil for stabilization) before it can be used as subgrade.

In terms of the above-mentioned reinforcement method using drainage boards with vacuum preloading, researchers have improved the materials or structures of drainage boards to alleviate clogging, improve drainage paths, and enhance the reinforcement effect. In terms of structural improvements, studies found that appropriately reducing the width and spacing of drainage boards, as well as the combined use of lateral and vertical drainage boards, could improve drainage efficiency when the same number of boards were used [16,17]. In terms of material improvements, studies found that modified fiber drainage boards made from treated waste fibers and non-filter membrane straw drainage bodies using spiral straw filter layers exhibited higher drainage efficiency than standard plastic drainage boards [18,19]. Other studies made improvements to the vacuum preloading method to enhance drainage consolidation, such as alternating prefabricated radiant drain vacuum preloading, stacked prefabricated vertical drain vacuum preloading, and step vacuum preloading. These strategies improved vacuum preloading methods to reduce the maximum clogging range on either side of the drainage board and improve the reinforcement effect [20,21,22,23]. In addition, studies combined vacuum preloading with other drainage consolidation methods to delay drainage board clogging, such as surcharge preloading, intermittent heating and electroosmosis [24,25,26,27]. These improvements to vacuum preloading or to the drainage boards used in vacuum preloading effectively increased the drainage efficiency of the boards and reinforcement range of high-water-content soil foundations. However, most of the above studies focus on optimizing a single improvement direction (such as increasing the drainage efficiency of the drainage boards, increasing the effective range or depth of the drainage boards, or increasing the strength of the drainage boards, etc.) and therefore are somewhat limited in balancing the mechanical properties of the drainage boards with anti-clogging.

In this study, three new types of anti-clogging plastic drainage boards were developed with different structures by improving the structure of the filter membrane in the drainage board. Permeability tests were conducted to select the optimal structure. Using the selected model, an in-lab vertical draining test was carried out on soda-residue-stabilized soil with high water content to observe changes in water content, vacuum pressure, surface settlement, vane shear strength, and the pore water pressure of the soil with drainage board insertion depth. The anti-clogging plastic drainage board in this study not only reduces clogging in high-water-content soil but also improves the mechanical properties of the drainage board, providing a reference for its application in practical engineering.

## 2. Materials and Methods

### 2.1. Soda Residue and Drainage Boards

#### 2.1.1. Physical and Mechanical Properties of Soda Residue

**Figure 1:**
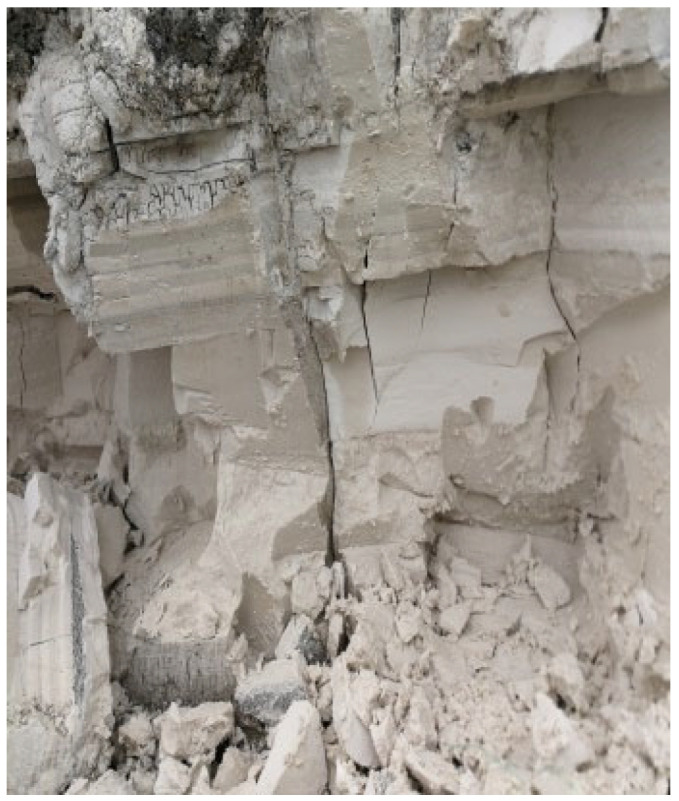
Soda residue in a natural state.

The soda residue used in this study was collected from a site in the Binhai District of Tianjin, China. In its natural state, soda residue appears as a milky white solid, with CaCO_3_ as the main chemical component. It typically consists of a substance with high structural integrity and becomes loose after air drying, as shown in Figure 1.

A sieve test was performed on natural-state soda residue to obtain the particle size distribution (Table 1).

As shown in Table 1, the particle size of the soda residue was largely in the 0.074–0.010 mm range, consisting primarily of cohesive silty sand.

The basic physical properties and mechanical parameters of soda residue were obtained from a series of indoor tests, as shown in Table 2 and Table 3.

As shown in Table 2 and Table 3, soda residue has extremely high water content and porosity in its natural state, resulting in low bulk density. Furthermore, the plasticity index of soda residue is 48.9, which is much higher than 17, indicating that it is a clayey soil.

#### 2.1.2. Structural Improvement of Plastic Drainage Boards

**Figure 2:**
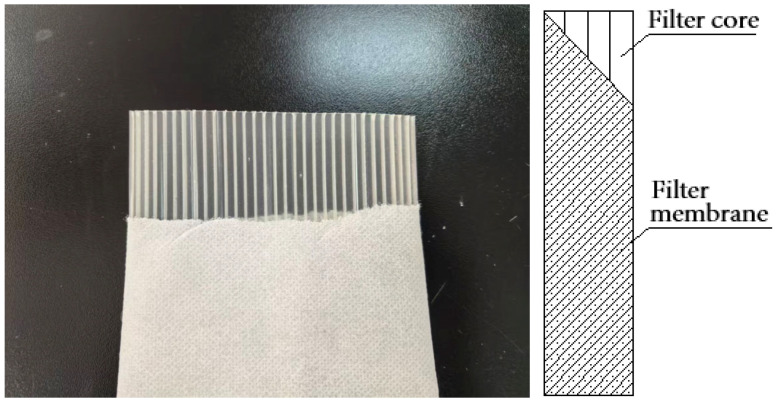
Standard plastic drainage board and its structure.

In this study, the filter membrane + filter core structure of standard plastic drainage boards (commonly used SPB-100 available on the market, Figure 2) was improved. The specifications of SPB-100 series are provided in Table 4.

Based on the standard model, the structure was improved to yield three types of modified plastic drainage boards (Type X, Type Y, and Type Z with enhanced hydraulic conductivity and bending resistance).

**Figure 3:**
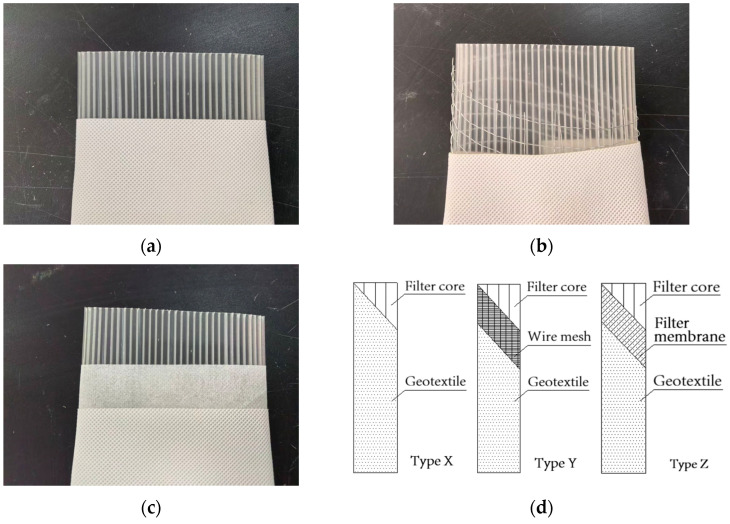
Three types of modified plastic drainage boards and their schematics (**a**) Type X; (**b**) Type Y; (**c**) Type Z; (**d**) schematics of three types of modified plastic drainage boards.

Type X board was fabricated by removing the original filter membrane of the standard drainage board and rewrapping a layer of geotextile fabric (surface mass 80 ± 10 g/m^2^, O_95_ effective pore size 110 ± 30 µm) around the filter core. Thus, the structure of the Type X board consisted of a filter core + geotextile fabric (from inside to outside). Compared to the filter membrane, the geotextile fabric possessed higher hydraulic conductivity. While increasing the overall drainage efficiency of the board, it also effectively prevented soda residue particles from entering the interior of the drainage board and avoid clogging caused by the attachment of particles to the core. The Type X plastic drainage board and its structure are shown in Figure 3a,d.

The Type Y board was made by removing the original filter membrane of the standard board, wrapping a layer of wire mesh around the filter core, and then wrapping a layer of geotextile fabric (same as geotextile fabric in Type X drainage board) outside the wire mesh. Thus, the structure of the Type Y board consisted of a filter core + wire mesh + geotextile fabric (from inside to outside). The role of the wire mesh was to increase the overall bending resistance of the drainage board and prevent it from bending. The Type Y board and its structure are shown in Figure 3b,d.

The Type Z drainage board was fabricated by wrapping a layer of geotextile fabric (same as geotextile fabric in Type X drainage board) around the filter membrane of the standard drainage board. Thus, the structure of the Type Z plastic drainage board consisted of a filter core + filter membrane + geotextile fabric (from inside to outside). The Type Z drainage board and its structure are shown in Figure 3c,d.

The YT010 geosynthetic material vertical permeability tester (Changzhou No.1 Textile Equipment Co., Ltd., Changzhou, China), the YG026MB-250 multi-functional electronic fabric tensile tester (Changzhou No.1 Textile Equipment Co., Ltd., Changzhou, China) and the TMC-2010electronic universal tester (Sinotest Equipment Co., Ltd., Changchun, China) were used to the permeability tests, filter membrane and composite tensile strength tests and effective bending strength tests on standard plastic drainage boards and X-, Y- and Z-type plastic drainage boards, respectively. The hydraulic conductivity, filter membrane tensile strength and overall tensile strength of the four types of plastic drainage boards are shown in Table 5.

**Figure 4:**
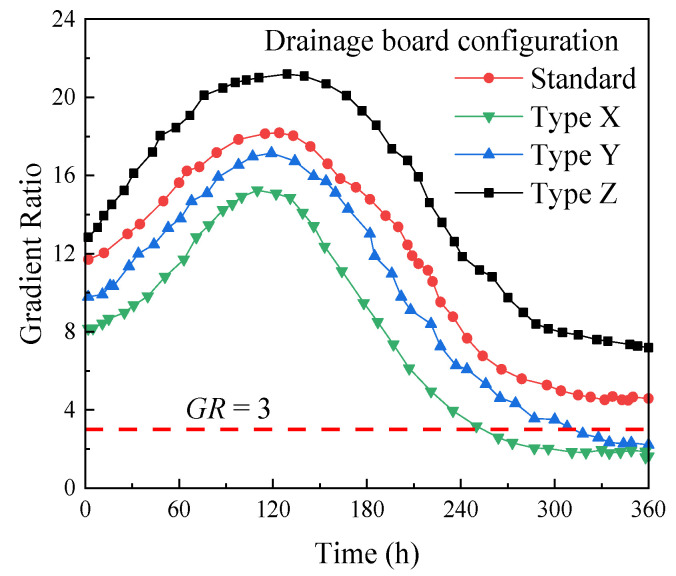
Hydraulic gradient Ratios of Four Types of Drainage Boards over Time.

Hydraulic gradient ratio tests were conducted on standard plastic drainage boards and X-, Y-, and Z-type plastic drainage boards using an MTSY-13 geosynthetic hydraulic gradient ratio tester (Tianjin Meitesi Testing Machine Factory, Tianjin, China), with the hydraulic gradient *i* range from 5.7 to 20. The hydraulic gradient *i* = 11 was selected during the test. The test duration was 360 hours. The hydraulic gradient ratio of the four types of drainage boards as a function of the test time was obtained, as shown in Figure 4.

As shown in Figure 4, the final hydraulic gradient ratio of type X and type Y plastic drainage boards *GR* < 3, while the final hydraulic gradient ratio of standard plastic drainage boards and type Z plastic drainage boards *GR* > 3, indicating that the clogging phenomenon of type X and type Y plastic drainage boards is not obvious.

As can be seen from Table 5 and Figure 4, among the four types of drainage boards, type X and type Y drainage boards have higher hydraulic conductivities and less clogging phenomenon. Furthermore, the wire mesh in type Y drainage boards increases its overall rigidity. Therefore, type Y drainage boards are preferred, and all subsequent experiments are based on type Y drainage boards.

### 2.2. Experimental Apparatus and Methods

**Figure 5:**
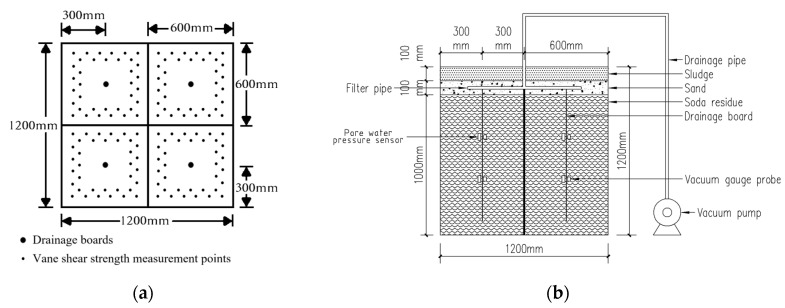
Schematic diagram of the experimental apparatus (**a**) Plan; (**b**) Perspective.

The experimental apparatus used in this study consisted of four parts: a vacuum pressure system, a water circulation system, a soda residue settlement system, and a monitoring and measurement system. A schematic diagram of the experimental apparatus is shown in Figure 5.

The on-site field condition in this study is an area with a size of 50 m × 50 m, the insertion depth of drainage boards is 6m and the spacing is 1 m, meaning the effective radius of each drainage plate is 0.71 m. In this test, the geometric similarity ratio *λ* = 6, which corresponds to the depth of the drainage boards is 1m and the effective radius of the drainage boards is 120 mm.

**Figure 6:**
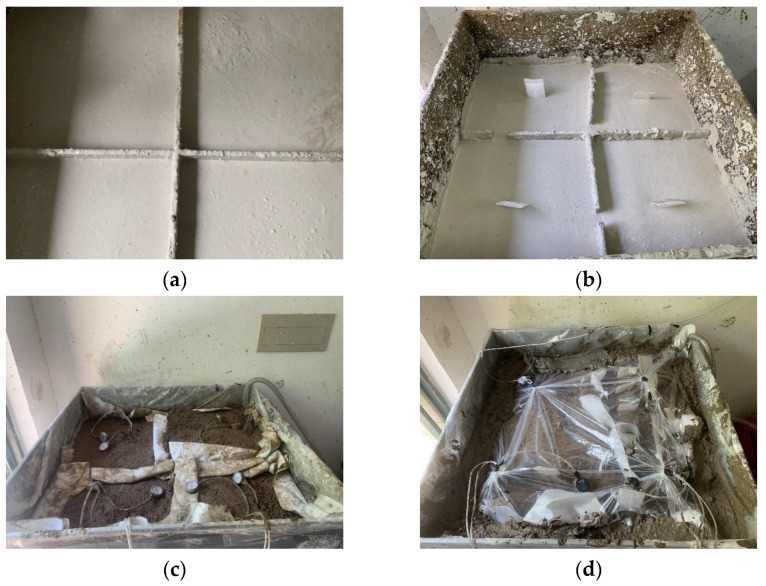
Test setup (**a**) Filling soda residue specimen; (**b**) Installing the drainage board; (**c**) Laying sand cushion and routing pipelines; (**d**) Laying sludge layer.

The mold used in the test consisted of a cubic box with a side length of 1.2 m. The interior of the box was partitioned with wooden boards into four equal compartments, with proper sealing and waterproofing treatment to ensure their complete separation (to study the effect of drainage board insertion depth), settlement measurement scales were affixed around the perimeter of each area to measure the surface settlement of the soda residue during the experiment. Soda residue with an initial water content of 230% was prepared by adding water to the natural-state soda residue, and the soda residue was poured into the mold (depth of 1 m) after thorough mixing, as shown in Figure 6a.

The bottom of the drainage board was wrapped with geotextile. The drainage board was slowly inserted into the soda residue to the target depth (the insertion depths were 25, 50, 75, and 100 cm in the four compartments). A vacuum gauge and pore water pressure sensor were installed at depths of 30 and 60 cm below the upper surface of soda residue. The geotextile was cut into suitable sizes, with openings made in the middle to allow the wires of the drainage board, vacuum gauge, and pore pressure meter to pass through. Subsequently, the geotextile was laid evenly in the four compartments of the mold, as shown in Figure 6b.

The drainage pipe was connected to the vacuum pump (6 L/min) and placed on top of the geotextile, and a 10 cm thick sand layer was laid. Multiple small holes were then drilled into the drainage pipe with an electric drill to improve the drainage performance, and the pipe was wrapped with geotextile to prevent sand from entering and clogging the pipeline. The connection between the drainage pipe and vacuum pump was sealed to ensure airtightness of the vacuum. The wires of the vacuum gauge and pore water pressure sensors were pulled out, as shown in Figure 6c.

Geotextile and plastic film were laid on top of the sand layer, followed by a 10 cm thick layer of sludge. The sludge was further covered with plastic film to ensure airtightness, as shown in Figure 6d.

Before the experiment began, the vacuum pump was started to conduct an air-tightness test. With the maximum vacuum pressure achieved by the system consisting of 97 kPa (gauge), the vacuum pressure was maintained at above 95 kPa (gauge), indicating good overall airtightness. Settlement, vacuum pressure, and pore water pressure were recorded at regular intervals. Each time after the pump was stopped, the water content and vane shear strength in the four compartments of the mold were measured. Data were collected every 4 h during the first 72 h of the experiment, after which, as the drainage volume decreased and settlement became less obvious, data were collected every 24 h. The experiment was stopped after 480 h.

## 3. Results

### 3.1. Water Content Change

**Figure 7:**
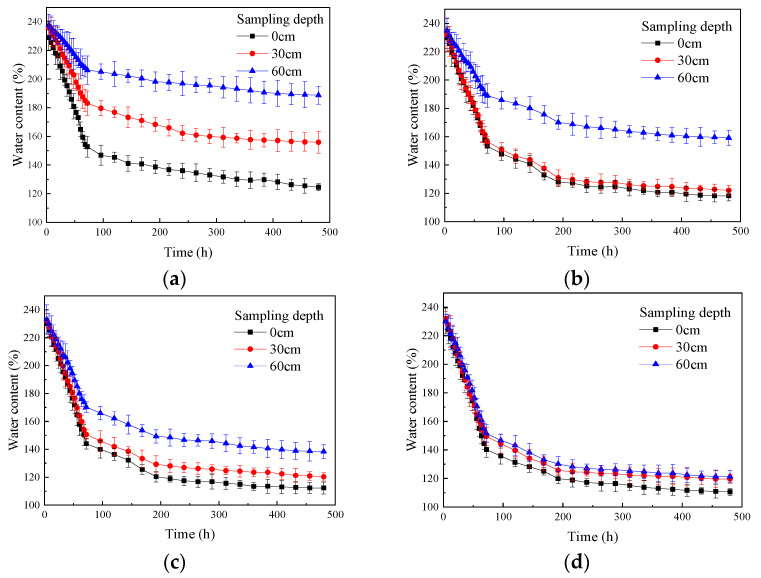
Change in water content of the soda residue over time (**a**) 25 cm; (**b**) 50 cm; (**c**) 75 cm; (**d**) 100 cm drainage board insertion depth.

Based on the test results, plots were made to demonstrate the change in water content of the soda residue at the surface and various depths below the surface over time under different drainage board insertion depths (Figure 7).

As shown in Figure 7, for different drainage board insertion depths, the water content of soda residue at the surface and various depths below the surface decreased monotonically as the test proceeded. The water content change exhibited a significant inflection point at around 72 h into the test. During the first 72 h, the rate of water content reduction was relatively fast, while after 72 h, the rate of reduction gradually slowed down and eventually stabilized at a certain level. Figure 7 also shows that at the same depth, water content reduction increased with an increase in drainage board insertion depth. For the same insertion depth, the water content increased with sampling depth, as detailed below.

1. Initially, the content of free water in the soda residue was relatively high, and during the early stage of the test, a large amount of free water was extracted. As the test progressed, the free water content decreased, while bound water was generally not extracted.
2. As water in the pores between the soda residue particles was drawn out, the pores were gradually compressed, the drainage channels became narrower, the seepage rate slowed down, and thus the rate of water content reduction decreased.
3. Pore compression hinders the effective downward transmission of negative pressure, weakening the drainage driving force (negative pore water pressure).

**Figure 8:**
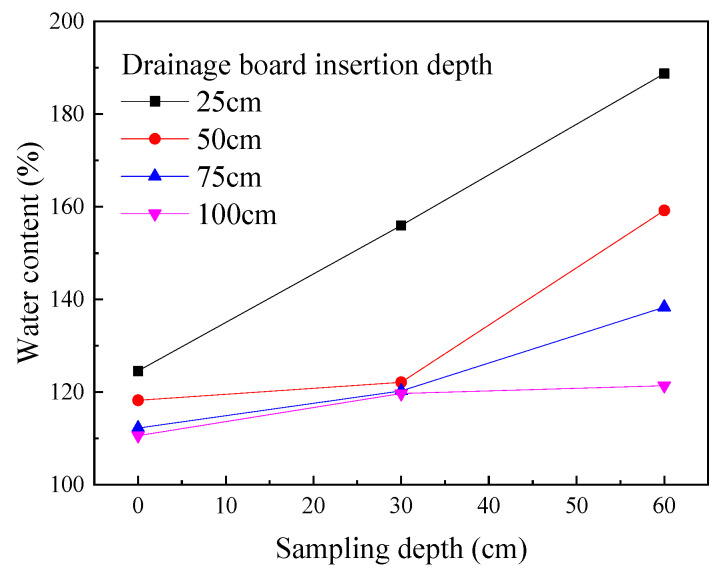
Final water content of soda residue vs. sampling depth.

Based on Figure 7, a curve was plotted demonstrating the relationship between the final water content of the soda residue at the end of the test and the sampling depth under different drainage board insertion depths (Figure 8).

As shown in Figure 8, the final water content of the soda residue at the surface decreased with increasing drainage board insertion depth, indicating that insertion depth not only affected the lower layers of the soda residue but also contributed to the drainage reinforcement of the upper layers. Furthermore, as the insertion depth increased from 25 to 50 cm, the final water content at a depth of 60 cm also decreased, which suggested that the effective range of the drainage board extended not only to the sides of the board but also had a drainage reinforcement effect on the soda residue below the bottom of the board.

### 3.2. Vacuum Pressure Change

The vacuum gauges (Shanghai Qingxun Instrument Co., Ltd., Shanghai, China) used in this test have a range of 0 to −100 kPa.

**Figure 9:**
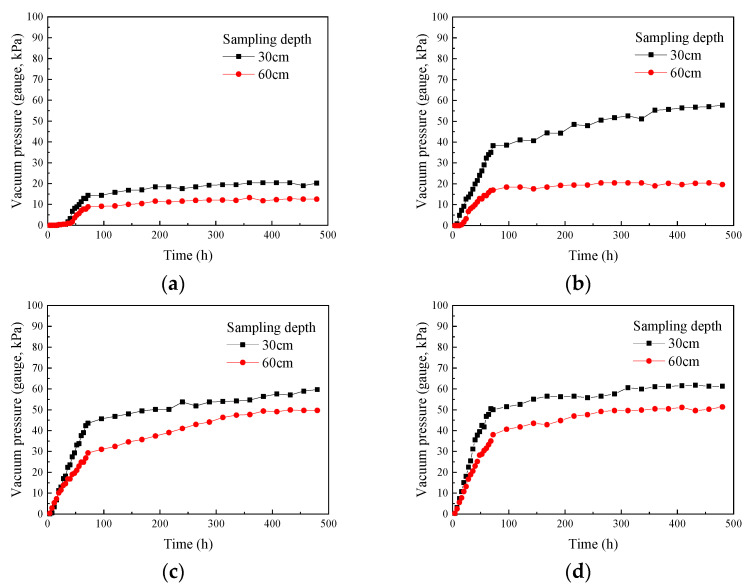
Change in vacuum change in soda residue specimen over time (**a**) 25 cm; (**b**) 50 cm; (**c**) 75 cm; (**d**) 100 cm drainage board insertion depth.

Based on the test results, plots were made to show the change in vacuum pressure over time at various depths below the surface of the soda residue under different drainage board insertion depths (Figure 9).

As shown in Figure 9, for different drainage board insertion depths, the vacuum pressure at depths of 30 and 60 cm below the surface in the soda residue gradually increased over the course of the test, indicating that a vacuum environment was progressively established. The vacuum pressure change showed a significant inflection point at around 72 h into the test. During the first 72 h, the vacuum pressure increased rapidly and exhibited an approximately linear relationship with time. The slope of the vacuum pressure-time curve at 30 cm deep was greater than at 60 cm, indicating a faster increase in vacuum pressure at 30 cm. This suggested a lag in the transmission of the negative pressure formed by the vacuum within the soda residue. After 72 h, the rate of increase in vacuum pressure gradually slowed down and stabilized at a certain level.

Comparing Figure 9c,d, we observed that in the later stages of the test, the trends in vacuum pressure change were nearly identical in both cases. However, in the early stage of the test, the rate of vacuum pressure increase was faster when the drainage board was inserted to a depth of 100 cm (Figure 9d), compared to 75 cm (Figure 9c), indicating that increasing the insertion depth accelerated the transmission speed of vacuum pressure within the soda residue.

### 3.3. Surface Settlement

**Figure 10:**
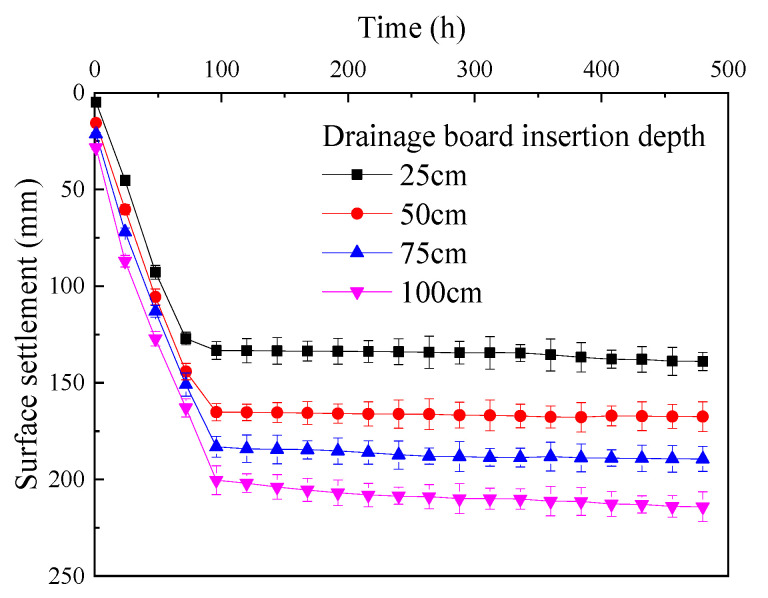
Change in the surface settlement of soda residue over time.

Based on the test results, curves were plotted to show the change in surface settlement of the soda residue over time under different drainage board insertion depths (Figure 10).

As shown in Figure 10, for different drainage board insertion depths, the surface settlement monotonically increased as the test proceeded. The surface settlement change exhibited a clear inflection point at around 72 h into the test. During the first 72 h, the rate of surface settlement increase was relatively fast, while after 72 h, the surface settlement rapidly stabilized, and as the test continued, the surface settlement remained essentially unchanged. Figure 10 also shows that the insertion depth of the drainage board had a significant impact on the surface settlement of the soda residue, where the greater the insertion depth, the more substantial the increase in surface settlement for the same test duration.

Due to the presence of geotextile on the surface of the drainage board, only a small number of fine soda residue particles was extracted during vacuum preloading. The main cause of settlement in the soda residue was the discharge of free water. Therefore, the increase in surface settlement was strongly correlated with the reduction in water content of the soda residue. The reasons for the gradual slowing of settlement were consistent with those causing the gradual slowing of the water content reduction.

### 3.4. Vane Shear Strength

Two types of vanes were used in the experiment: one with a size of 50.8 mm × 101.6 mm (maximum range 8.125 kPa) and the other with a size of 25.4 mm × 50.8 mm (maximum range 65 kPa). Each vane shear test was performed at a different measuring point, and the vane shear strength measurement points are shown in Figure 5a.

**Figure 11:**
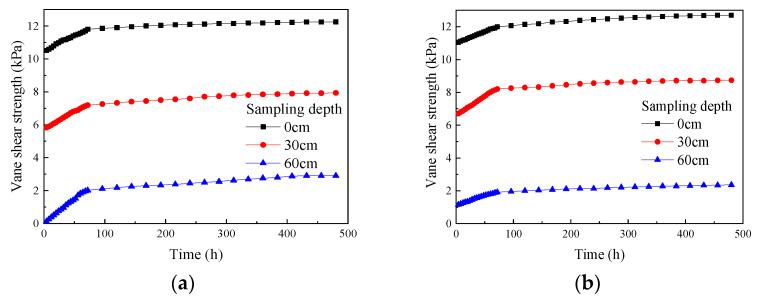

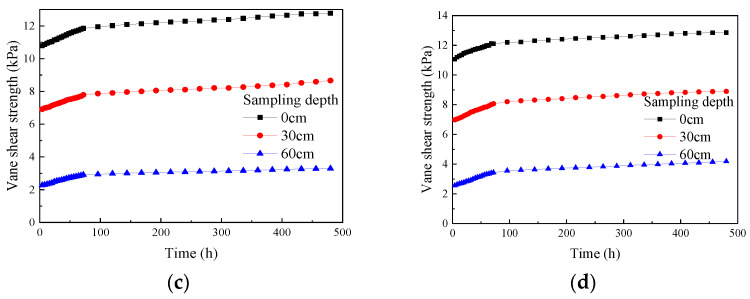
Change in the vane shear strength of soda residue over time (**a**) 25 cm; (**b**) 50 cm; (**c**) 75 cm; (**d**) 100 cm drainage board insertion depth.

According to the test results, plots were made to show the change in vane shear strength of the soda residue at the surface and at various depths below the surface over time under different drainage board insertion depths (Figure 11).

As shown in Figure 11, at different drainage board insertion depths, the vane shear strength of the soda residue at the surface and various depths below the surface increased monotonically as the test proceeded. The vane shear strength variation showed a clear inflection point at around 72 h into the test. During the first 72 h, the vane shear strength increased rapidly and exhibited an approximately linear relationship with time. Meanwhile, after 72 h, the growth rate of the vane shear strength gradually slowed, and the strength remained approximately linear relative to time, only with a reduced slope. However, unlike the variation in water content, vacuum pressure, and surface settlement with time, the vane shear strength kept increasing without an obvious plateau by 480 h, demonstrating that the increase in strength lagged behind the decrease in water content (i.e., rearrangement of soda residue particles lagged behind the extraction of pore water between particles in terms of time). In addition, Figure 11 shows that for the same insertion depth, the vane shear strength decreased with increasing sampling depth, as detailed below.

1. As pointed out in Section 3.1, the water content increased with increasing sampling depth. The higher water content in the lower soil layers led to greater pore water pressure, reducing the effective stress in the lower soil and resulting in lower strength.
2. As detailed in Section 3.2, the vacuum pressure in the lower layers of soda residue was lower. The lower vacuum pressure in the lower soil layers led to smaller absolute values of negative pore water pressure formed during the vacuum preloading process, further reducing the effective stress in the lower soil and resulting in lower strength.
3. The lower water content and higher vacuum pressure in the upper soil promoted the rearrangement of soda residue particles, increasing the compactness, increasing the contact area between the particles, increasing the internal friction angle, and significantly improving the strength.

**Figure 12:**
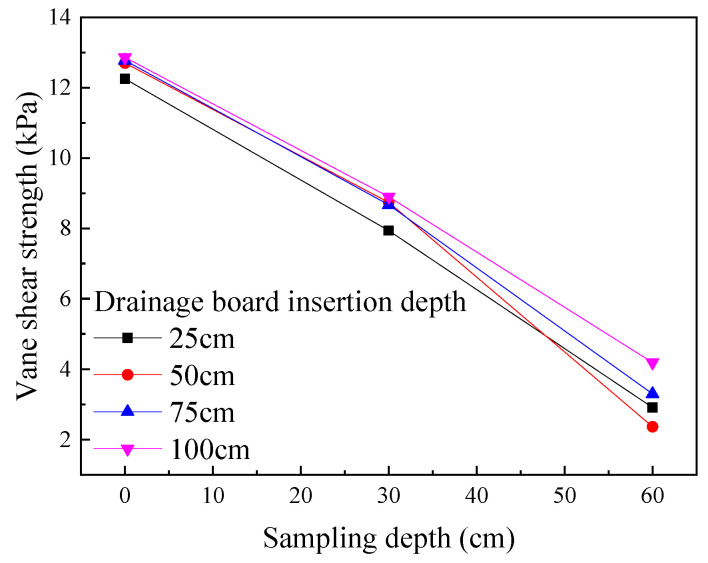
Final vane shear strength of soda residue vs. sampling depth.

As shown in Figure 11, a plot was made showing the relationship between the final vane shear strength of soda residue at the test conclusion and sampling depth under different drainage board insertion depths (Figure 12).

As shown in Figure 12, the insertion depth of the drainage board exerted an influence on the final vane shear strength of soda residue. At the same sampling depth, the greater insertion depth led to slightly higher vane shear strength.

### 3.5. Pore Water Pressure

The pore water pressure sensors used in this test have a range of 0 to −100 kPa.

**Figure 13:**
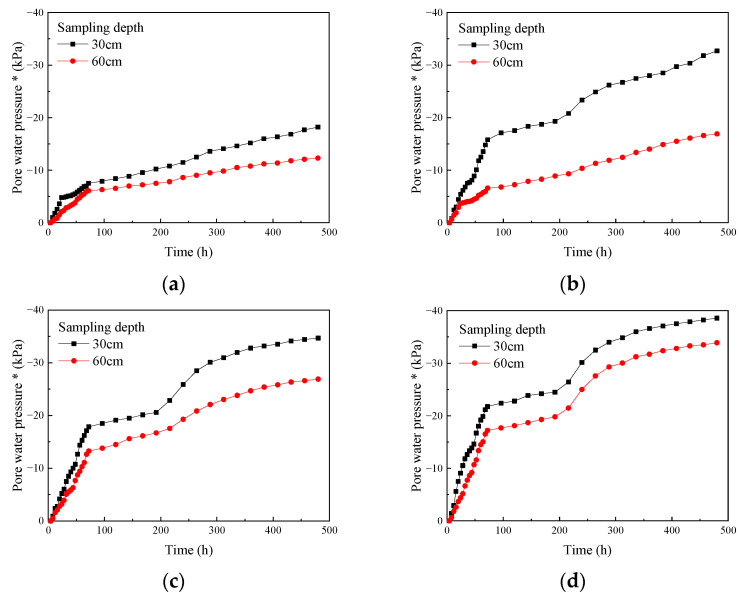
Change in pore water pressure of soda residue over time (**a**) 25 cm; (**b**) 50 cm; (**c**) 75 cm; (**d**) 100 cm drainage board insertion depth. * Vertical axis (pore water pressure) below 0 means vacuum.

Based on the test results, plots were made to show the change in pore water pressure over time at various depths and under different insertion depths (Figure 13).

As shown in Figure 13, for different drainage board insertion depths, the pore water pressure at various depths below the surface of the soda residue increased monotonically with test progression and exhibited a typical double-platform shape. This trend became more evident as the insertion depth increased. During the first 72 h of the test, the absolute value of the pore water pressure increased rapidly. Meanwhile, from 72 to 240 h, the growth rate of the absolute value of pore water pressure gradually slowed down, and the pore water pressure entered the first stable stage, and after 240 h, the absolute value increased again. However, as the test continued, the rate of increase in this absolute value gradually slowed down, and the pore water pressure entered the second stable stage. No clear inflection point was observed during the second increase, and the absolute value of pore water pressure continued to increase slowly until the end of the test (480 h). Figure 13 also shows that at the same depth below the surface, the absolute value of pore water pressure increased with increasing insertion depth. For the same insertion depth, the absolute value of pore water pressure decreased with increasing sampling depth, as detailed below.

Terzaghi’s (1943) [28] effective stress principle:

*σ*′ = *σ* − *u*, (1)

Under vacuum conditions (*u* < 0), we have:

*σ*′ = *σ* + |*u*|, (2)

From Equation (2), the increase in the absolute value of pore water pressure |*u*| consists of two parts: a decrease in total stress *σ* and an increase in effective stress *σ*′.

In the first 72 h after the start of the experiment, the water content of the soda residue decreases rapidly (Figure 7), the self-weight of the soda residue decreases rapidly, and the total stress (self-weight stress) *σ* of the soda residue decreases rapidly. At the same time, the strength of the soda residue increases (Figure 11), which means that the effective stress *σ*′ of the soda residue also increases. The decrease in total stress *σ* and the increase in effective stress *σ*′ together lead to the increase in the absolute value of pore water pressure |*u*| of the soda residue.

From 72 h to 240 h during the experiment, the rate of decrease in the water content of the soda residue was slow, as were the rates of decrease in the self-weight of the soda residue and the total stress (self-weight stress) *σ* of the soda residue. However, since the strength of the soda residue continued to increase, this meant that the effective stress *σ*′ of the soda residue also continued to increase. At this time, the increase in the absolute value of the pore water pressure |*u*| of the soda residue was mainly caused by the increase in the effective stress *σ*′.

In summary, the first stage of the pore water pressure (absolute value)-time curve growth is mainly attributed to the decrease in total stress and the increase in effective stress, while the second stage growth is mainly attributed to the increase in effective stress.

**Figure 14:**
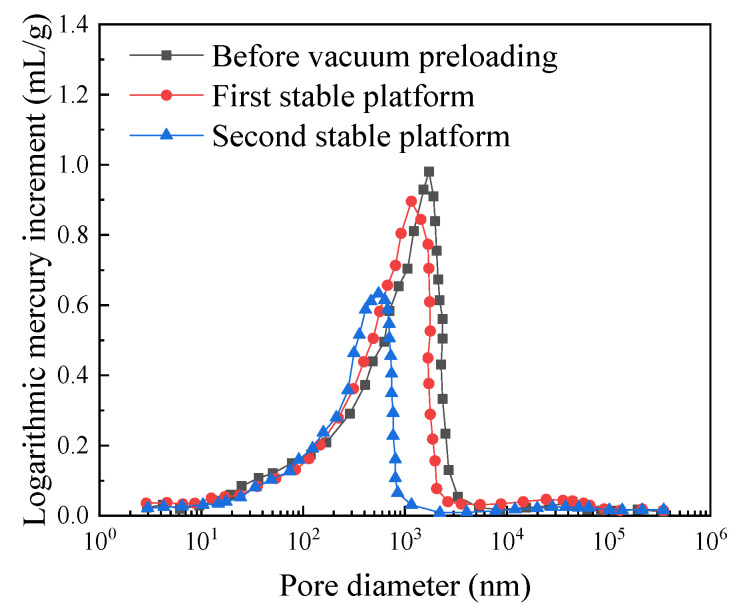
Pore volume (logarithmic mercury ingress increment) of pores with different diameters in soda residue.

Mercury intrusion porosimetry (MIP) tests were conducted on the soda residue before vacuum preloading, during the first stable plateau stage and at the second stable plateau stage to study the changes in the pore structure of the soda residue under the two stable plateaus. The experimental results are shown in Figure 14.

As shown in Figure 14, compared with before vacuum preloading, the pore diameter and pore volume (logarithmic mercury increment) of the soda residue at the first stable plateau decreased only slightly; compared with the first stable plateau, the pore diameter and pore volume (logarithmic mercury increment) of the soda residue at the second stable plateau decreased significantly. This indicates that the second increasing stage of the pore water pressure (absolute value) of the soda residue is mainly due to the decrease in pore size and pore volume, which verifies the above analysis.

### 3.6. Total Drained Water Mass

**Figure 15:**
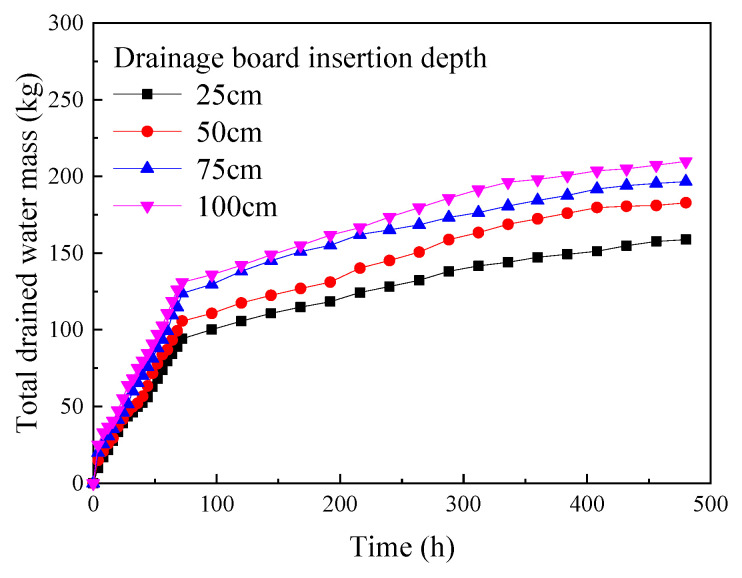
Total drained water mass.

Based on the experimental results, the curves showing the change in the total mass of water discharged during the experiment with the time of the experiment under different drainage board insertion depths are plotted, as shown in Figure 15.

**Figure 16:**
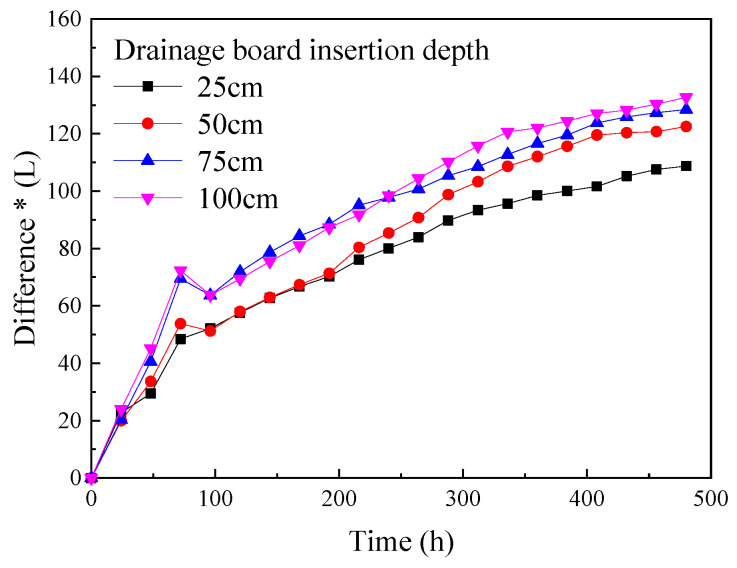
Difference between the reduction in soda residue volume and the total drainage volume. * Shows the total water drainage volume minus the reduction in the volume of soda residue.

As shown in Figure 15, for different depths of the drainage board insertion, the total drainage mass monotonically increases with the progress of the experiment. The total drainage mass change curve shows a clear inflection point after about 72 hours of experimentation: in the first 72 hours after the start of the experiment, the total drainage mass increases rapidly, and the total drainage mass is approximately linearly related to the experiment time; after 72 hours, the rate of increase of the total drainage mass gradually slows down, and the total drainage mass is still approximately linearly related to the experiment time, but the slope decreases. Moreover, the total drainage mass continues to increase slowly as the experiment progresses, the total drainage mass kept increasing without obvious plateau by 480 h, this pattern is similar to the change in the vane shear strength of soda residue over time described in Section 3.4, indicating that the total drainage mass also has a certain lag relative to the decrease in the water content. Figure 16 shows the difference between the total volume reduction in soda residue and the volume of drained water, plotted from Figure 10 (surface settling amount) and Figure 15.

As shown in Figure 16, at the beginning of the experiment, the total drainage volume was significantly greater than the volume of soda residue reduction, meaning that the volume reduction in soda residue lagged behind the drainage. As the experiment progressed, the growth of this difference gradually slowed down, meaning that the total drainage volume and the volume reduction in soda residue gradually reached equilibrium.

Combining the conclusions of Section 3.1 and Section 3.4, and this section, it can be concluded that at the beginning of vacuum preloading, the decrease in the water content of the soda residue occurs first. The increase in total drainage volume and the increase in the vane shear strength lag behind the decrease in the water content of the soda residue, while the settlement (volume reduction) of the soda residue lags behind the decrease in the water content. In summary, from the start of the experiment, the total drainage volume has been increasing. In the early stage of the experiment, the increase in total drainage volume corresponds to the decrease in the water content of the soda residue, but at this time, the soda residue particles have not yet rearranged, and the volume reduction in the soda residue (relative to the increase in total drainage volume) is not significant. In the later stage of the experiment, the increase in total drainage volume corresponds to the rearrangement of the soda residue particles (at which time the decrease in the water content of the soda residue also occurs simultaneously), and the volume reduction in the soda residue (relative to the increase in total drainage volume) is significant. These two drainage stages correspond to the two growth stages of the double plateau curve of pore water pressure in Section 3.5.

## 4. Discussion

### 4.1. Analysis of the Strength Growth Mechanism of Soda Residue

**Figure 17:**
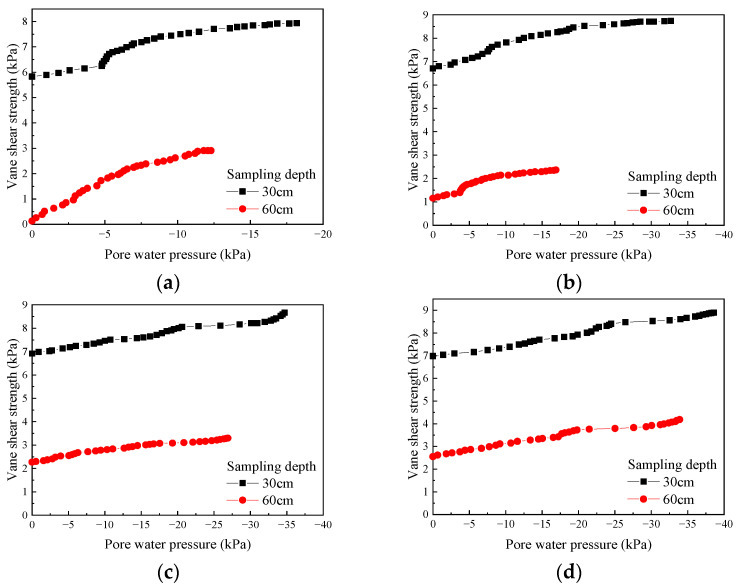
Relationship between the vane shear strength and pore water pressure of soda residue (**a**) 25 cm; (**b**) 50 cm; (**c**) 75 cm; (**d**) 100 cm drainage board insertion depth.

To further explore the strength development mechanism of soda residue, plots were made based on Figure 11 and Figure 13 to show the relationship between the vane shear strength and pore water pressure of soda residue under different drainage board insertion depths (Figure 17).

As shown in Figure 17, at the same drainage board insertion depth and same sampling depth, the vane shear strength increased with the absolute value of pore water pressure. At the same drainage board insertion depth and same pore water pressure, the vane shear strength decreased with increasing sampling depth. This indicated that the strength of soda residue was jointly determined by pore water pressure and effective stress (the sampling depth affects the effective stress). This also validated the conclusion in Section 3.4, indicating that the effective stress of the upper soil was greater than that of the lower soil, and the conclusion of Section 3.5, where the decrease in total stress and increase in effective stress together led to an increase in the absolute value of negative pore water pressure.

### 4.2. Verification of the Evolution of Pore Water Pressure

**Figure 18:**
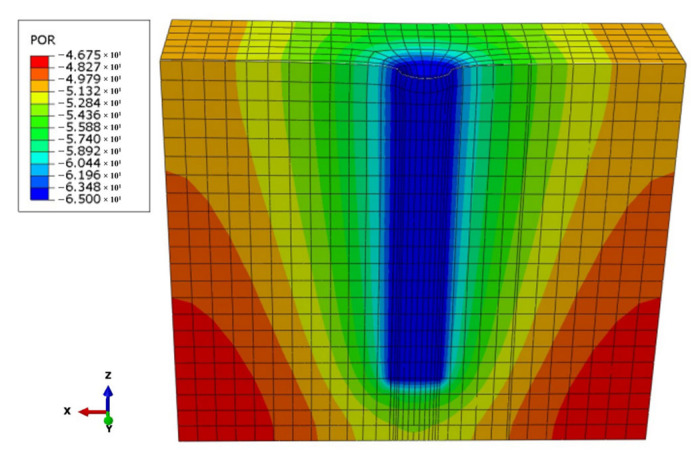
Pore water pressure cloud map of soda residue (480 h).

**Figure 19:**
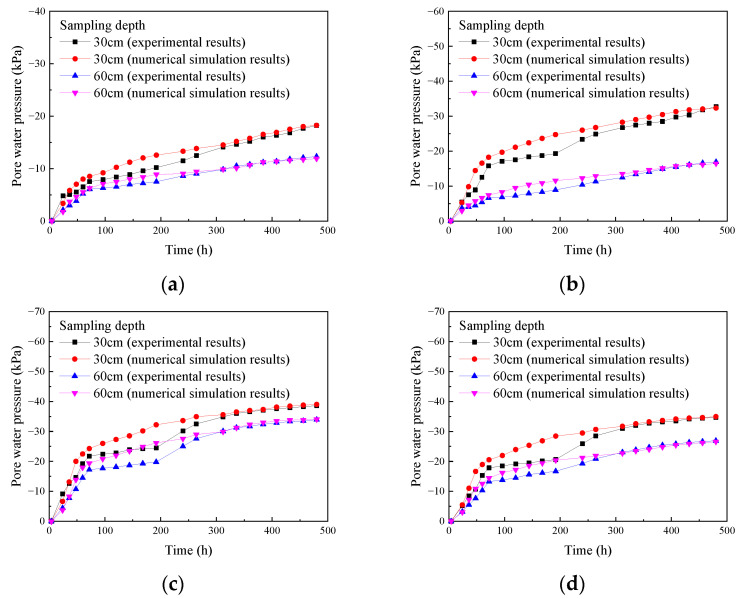
Comparison between the numerical simulation results and the experimental results of pore water pressure (**a**) 25 cm; (**b**) 50 cm; (**c**) 75 cm; (**d**) 100 cm drainage board insertion depth.

The density of the soda residue in Table 2, the elastic-plastic and permeability parameters of the soda residue in Table 3, and the elastic and permeability parameters of the drainage board in Table 5 were input into the finite element analysis software ABAQUS (2018) for analysis (Figure 18). A linear elastic model was used for the drainage board, and a Mohr-Coulomb model was used for the plastic model of the soda residue. The model dimensions were the same as those in the indoor model test in Section 2.2. Except for the top surface of the model, which is a permeable boundary, all other boundaries were impermeable. The model mesh side length was 5 cm, and the mesh type was C3D8P. The comparison between the numerical simulation results and the experimental results of pore water pressure is shown in Figure 19.

As shown in Figure 19, the numerical simulation results of pore water pressure are generally consistent with the experimental results. However, the numerical simulation results show a smoother trend in pore water pressure compared to the experimental results, exhibiting a double-plateau curve that is less pronounced than that in the experimental results. This is because the medium in the numerical simulation is continuous, and it is impossible to account for the increase in pore water pressure (absolute value) caused by the increase in effective stress due to the rearrangement of soda residue particles. This also validated the conclusion in Section 3.5, indicating that the decrease in total stress and increase in effective stress together led to an increase in the absolute value of negative pore water pressure.

### 4.3. Analysis of the Anti-Clogging Effect of the Novel Drainage Board

**Figure 20:**
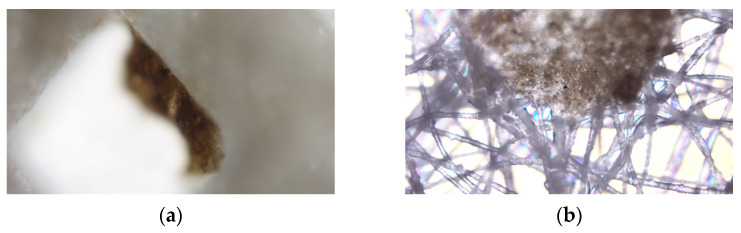
Optical micrographs of the filter membrane and geotextile after drainage (**a**) filter membrane; (**b**) geotextile.

The clogging of the drainage board filter membrane and geotextile after vacuum preloading was observed using an DP22 optical microscope (Olympus Corporation, Tokyo, Japan), with 10× objective lens and 10× eyepiece (100× magnification), as shown in Figure 20.

As shown in Figure 20, the filter membrane has larger pores, and soda residue particles are embedded in these pores. In contrast, the geotextile has smaller pores, and soda residue particles are trapped on its surface. This indicates that both the filter membrane and geotextile effectively block soda residue particles; however, prolonged use can lead to clogging of the filter membrane due to the embedding of soda residue particles. Furthermore, although the geotextile has smaller pores than the filter membrane, and the fibers constituting the geotextile also have smaller diameters, the overall porosity of the geotextile is similar to that of the filter membrane. Therefore, this does not affect water permeability efficiency.

### 4.4. Generalizing to Actual Dimensions

In Terzaghi’s one-dimensional consolidation theory, the consolidation coefficient *C_v_* is:

(3) $$C_{v}=\frac{K(1+e)}{a\gamma_{w}},$$

In the formula: *C_v_* is the consolidation coefficient; *K* is the permeability coefficient; *e* is the void ratio; *a* is the compressibility coefficient; *γ_w_* is the unit weight of water.

**Figure 21:**
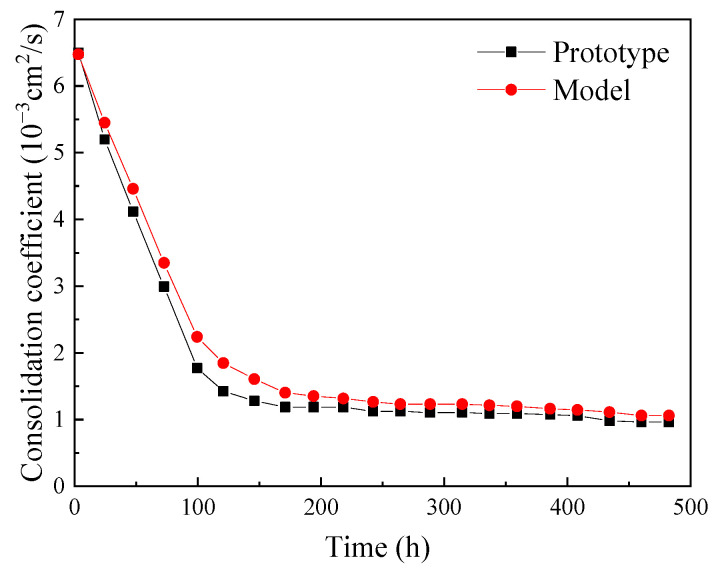
Consolidation coefficient of the prototype and model over time.

It is evident that the consolidation coefficient *C_v_* is not a constant value due to parameter changes during the vacuum preloading process (e.g., a decrease in void ratio *e*); however, for samples with the same permeability coefficient *K*, compressibility coefficient *a*, and initial porosity *e*_0_, the variation in their consolidation coefficient *C_v_* should be similar. In this study, the variation in the consolidation coefficient of the prototype and model over time is shown in Figure 21:

As shown in Figure 21, due to the different degrees of perturbation and boundary conditions between the prototype and the model, their consolidation coefficients are slightly different: the model’s consolidation coefficient is slightly larger than the prototype’s, indicating that the smaller model is more susceptible to perturbation and experiences more significant structural damage.

In Terzaghi’s one-dimensional consolidation theory, the consolidation time factor *T_v_* is:

(4) $$T_{v}=\frac{c_{v}t}{H^{2}},$$

That is:

(5) $$t=\frac{T_{v}}{c_{v}}H^{2},$$

In the formula: *t* is the consolidation time; *T_v_* is the consolidation time factor; *C_v_* is the consolidation coefficient; *H* is the equivalent drainage path length, proportional to similarity ratio *λ*.

Based on the geometric similarity of the consolidation curves, we have:

(6) $$T_{v}(prototype)=T_{v}(model),$$

From Figure 21, we can see that:

(7) $$C_{v}\left(prototype\right)\approx C_{v}(model),$$

Combining Formulas (5) to (7), we get:

(8) $$t\propto H^{2},$$

Further:

(9) $$t\propto \lambda^{2},$$

In the formula: *λ* is the similarity ratio.

In summary, when generalizing the research results to actual dimensions, all geometric scales need to be multiplied by the similarity ratio *λ*, and all time scales (e.g., the time to reach the same degree of consolidation) need to be multiplied by the square of the similarity ratio *λ*^2^.

## 5. Conclusions

### 5.1. Fundamental Conclusions

1. During the vacuum preloading process, the decrease in the water content of the soda residue occurs first, whereas the increase in total drainage volume and vane shear strength lags behind the reduction in water content, and the volume decrease in the soda residue further lags behind the increase in the total drainage volume.
2. During vacuum preloading, the increase in the pore water pressure (absolute value) of the soda residue is influenced by both the decrease in total stress and the increase in effective stress, exhibiting a typical double-plateau curve. The first stage of pore water pressure (absolute value) increase is mainly attributed to the decrease in total stress caused by the reduction in water content and self-weight of the soda residue, whereas the second stage is primarily governed by the increase in effective stress resulting from the reduction in pore volume and pore diameter.
3. The mechanical properties and anti-clogging performance of drainage boards are highly dependent on their structural configuration. Introducing a wire mesh between the filter core and the geotextile significantly enhances the tensile and bending strength of the drainage board without noticeably compromising its drainage performance.

### 5.2. Applied Conclusions

1. The type Y anti-clogging plastic drainage board (geotextile + wire mesh + filter core) exhibits the most balanced performance in terms of permeability, anti-clogging ability, tensile strength and bending strength and is suitable for vacuum preloading of soda residue with high water content.
2. The insertion depth of the drainage board significantly affects drainage efficiency, vacuum transmission rate, and strength development of the soda residue. The effective reinforcement range of the drainage board is not limited to the insertion depth but also extends below the bottom of the drainage board.
3. When extrapolating the research results to on-site field conditions, geometric parameters such as the effective drainage range (radius of influence of drainage boards), drainage depth and drainage board spacing should be scaled by the similarity ratio, whereas the consolidation time scale should be scaled by the square of the similarity ratio.

## Figures and Tables

**Table 1:** Particle size distribution of soda residue.

**Particle size** **(mm)**	>0.420	0.420~0.177	0.177~0.149	0.149~0.074	0.074~0.010	<0.010
**Mass ratio** **(%)**	1.20	1.92	2.10	4.50	80.10	10.18

**Table 2:** Basic physical properties of soda residue.

Natural Density	Relative Density	Water Content	Plastic Limit	Liquid Limit	Plasticity Index	Liquidity Index	Unit Weight	Dry Unit Weight	Void Ratio	Porosity	Saturation
*ρ*	*G*_s_	*ω*	*ω*_p_	*ω*_l_	*I*_p_	*I*_l_	*γ*	*γ*_d_	*e*	*n*	*S*_r_
(g/cm^3^)		(%)	(%)	(%)			(kN/m^3^)	(kN/m^3^)		(%)	(%)
1.19	2.33	216.40	87.4	136.3	48.9	2.6	12.21	3.86	4.73	82	100.00

**Table 3:** Elastic, plastic and permeability parameters of soda residue.

Compression Modulus	Cohesion	Internal Friction Angle	Hydraulic Conductivity
*E*_s_	*c*	*φ*	*κ*
(MPa)	(kPa)	(°)	(cm/s)
1.1	8.0	6.0	1.4 × 10^−5^

**Table 4:** Specifications of SPB-100 drainage boards.

Filter Membrane				Filter Membrane and Filter Core Composite	
Tensile Strength (Dry State)	Tensile Strength (After 24 h Immersion)	Hydraulic Conductivity (After 24 h Immersion)	Effective Pore Size (O_95_)	Tensile Strength (at 10% Elongation)	Longitudinal Flow Rate (Under 350 kPa Lateral Pressure)
25 N/cm	20 N/cm	6.6 × 10^−4^ cm/s	0.075 mm	1.5 MPa	30 cm^3^/s

**Table 5:** The hydraulic conductivities and tensile strengths of four types of drainage boards.

	Standard Plastic Drainage Board	Type X Plastic Drainage Board	Type Y Plastic Drainage Board	Type Z Plastic Drainage Board
Hydraulic conductivity	6.6 × 10^−4^ cm/s	1.7 × 10^−3^ cm/s	1.7 × 10^−3^ cm/s	3.9 × 10^−4^ cm/s
Filter membrane tensile strength (dry state)	31.3 N/cm	33.4 N/cm	37.1 N/cm	34.5 N/cm
Filter membrane tensile strength (after 24 h immersion)	20.2 N/cm	21.3 N/cm	25.3 N/cm	21.8 N/cm
Composite tensile strength (at 10% elongation)	1.50 MPa	1.60 MPa	2.03 MPa	1.88 MPa
Effective bending strength	38 kPa	38 kPa	75 kPa	38 kPa

## Data Availability

The original contributions presented in this study are included in the article. Further inquiries can be directed to the corresponding author.
